# Chemical Structure Identification in Metabolomics: Computational Modeling of Experimental Features

**DOI:** 10.5936/csbj.201302005

**Published:** 2013-03-01

**Authors:** Lochana C. Menikarachchi, Mai A. Hamdalla, Dennis W. Hill, David F. Grant

**Affiliations:** a Department of Pharmaceutical Sciences, University of Connecticut, 69 N Eagleville Rd, Storrs, CT 06269, United States; b Department of Computer Science & Engineering, University of Connecticut, 371 Fairfield Road, Unit 2155 Storrs, CT 06269, United States

**Keywords:** metabolomics, mass spectrometry, HPLC, QSPR, retention index, ion mobility

## Abstract

The identification of compounds in complex mixtures remains challenging despite recent advances in analytical techniques. At present, no single method can detect and quantify the vast array of compounds that might be of potential interest in metabolomics studies. High performance liquid chromatography/mass spectrometry (HPLC/MS) is often considered the analytical method of choice for analysis of biofluids. The positive identification of an unknown involves matching at least two orthogonal HPLC/MS measurements (exact mass, retention index, drift time etc.) against an authentic standard. However, due to the limited availability of authentic standards, an alternative approach involves matching known and measured features of the unknown compound with computationally predicted features for a set of candidate compounds downloaded from a chemical database. Computationally predicted features include retention index, ECOM_50_ (energy required to decompose 50% of a selected precursor ion in a collision induced dissociation cell), drift time, whether the unknown compound is biological or synthetic and a collision induced dissociation (CID) spectrum. Computational predictions are used to filter the initial “bin” of candidate compounds. The final output is a ranked list of candidates that best match the known and measured features. In this mini review, we discuss cheminformatics methods underlying this database search-filter identification approach.

## Introduction

Metabolomics focuses on the study of small molecules (usually < 1000 Da) produced by metabolic processes within a cell. These small molecules, called metabolites, encompass a broad range of compounds composed of a variety of chemical functional groups [1, 2]. The term “metabolome” is used to identify the collection of such small molecules in an organism [3]. It is estimated that a metabolome may comprise anywhere from 1000 – 200,000 distinct chemical compounds depending on the organism [1]. A typical human biofluid such as blood or urine may contain several hundreds to thousands of unique chemical compounds. For example, as of August 8, 2012, the human metabolome database (HMDB) [4] listed 942, 4651, and 468 compounds in urine, blood and cerebrospinal fluid respectively. The separation and concurrent identification of chemical structures in such complex mixtures often require multiple analytical techniques. The most commonly used analytical techniques include nuclear magnetic resonance (NMR), gas chromatography/mass spectrometry (GC/MS) and high performance liquid chromatography/mass spectrometry (HPLC/MS). Positive identification of an unknown involves matching at least two orthogonal experimental features with an authentic standard. In the case of NMR, a match against ^1^H NMR and ^13^C NMR or a match against a 2D NMR is considered sufficient for identification. In GC/MS and HPLC/MS, orthogonal experimental features may include retention index (RI) or retention time, accurate mass, isotope abundance pattern and a collision induced dissociation (CID) spectrum. When analyzing complex mixtures containing polar compounds, HPLC/MS is generally preferred over GC/MS as GC often requires sample derivatization. In metabolomics, HPLC/MS based identification is more frequently used compared to NMR due to its increased sensitivity.

**Figure 1:**
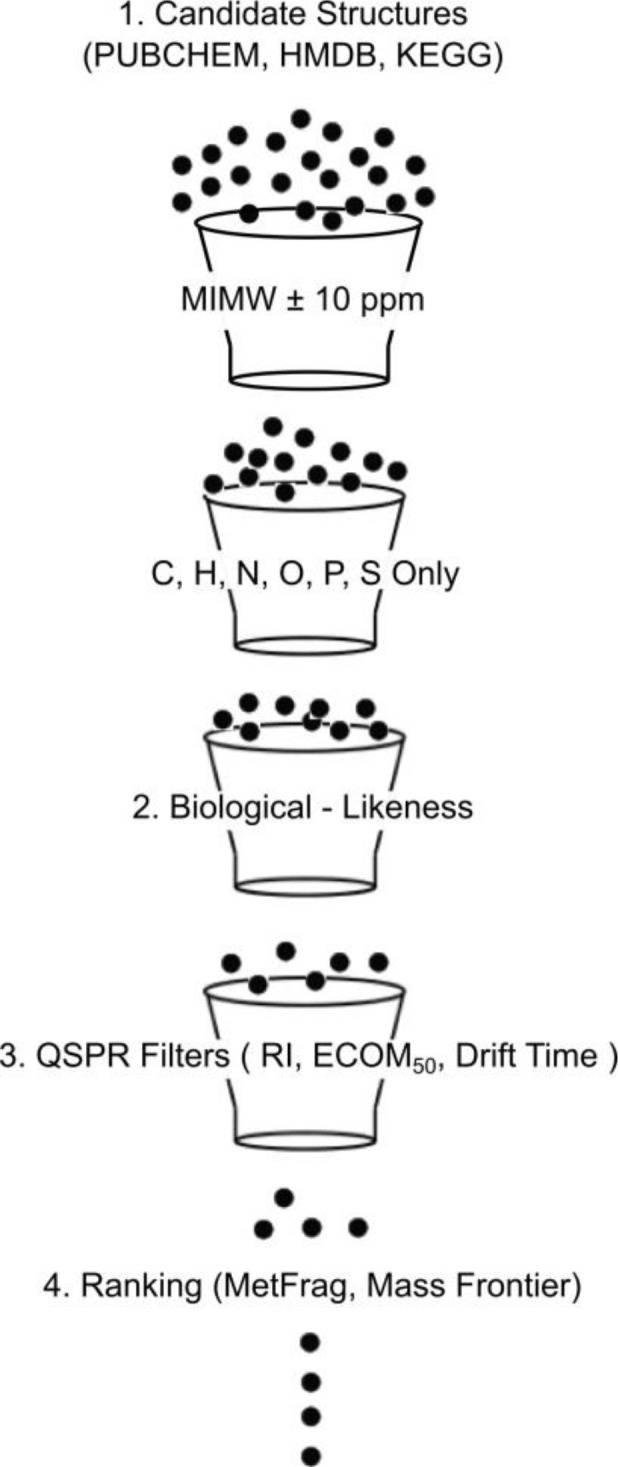
Filtering database candidates using simulated experimental data.

Regardless of the analytical method, the structural identification of unknowns is severely limited by the number of commercially available authentic standards available to any one lab. For HPLC/MS methods considered in this review, an alternative approach involves matching experimentally determined “features” (such as RI, mass spectrum etc.) with computationally simulated features for a set of compounds downloaded from a general chemical structure database such as PubChem [5–7] (Fig 1).

In this approach, a “bin” of candidate compounds matching an experimentally determined monoisotopic molecular weight (MIMW) (± mass accuracy of the instrument) is first retrieved from the database. If we assume that the sample is a mammalian biofluid (for example human urine or serum) the remaining compounds can then be filtered based on whether or not they contain only 6 allowed elements (C, H, N, O, P and S). Making the same assumption, the remaining compounds can then be filtered based on whether or not they are “biochemical” structures. For the remaining candidates, experimental measurements such as RI, ECOM_50_ (energy required to decompose 50% of a selected precursor ion in a CID cell) and drift time are modeled using quantitative structure-property relationships (QSPR). Candidate compounds whose predicted features lie outside the range of values allowed by the QSPR models are removed from the bin. For each of the remaining candidates, a simulated collision induced dissociation (CID) spectrum is calculated and matched against the experimental CID spectrum. Candidates are then ranked according to the number of predicted peaks matching the experimental peaks. All steps in the structure identification protocol described above can be performed with the free java based software package MolFind [8]. In the discussion that follows, each step of the aforementioned protocol will be discussed in detail. Topics are arranged according to the numbered steps in figure 1.

## 1. Candidate Structures

Database structure identification involves downloading a “bin” of potential candidate structures matching a MIMW (± mass accuracy of the instrument) from an online chemical structure database. Databases range from general chemical structure databases such as PubChem [9], ZINC [10] or ChemSpider [11] to specialized databases such as HMDB [4], DrugBank [12], or HumanCyc [13]. A list of freely accessible small molecule databases useful in metabolomics research is presented in Table 1.

**Table 1:** Freely accessible databases useful in metabolomics research.

Database	Type	# of cpds	Ref
PubChem	General chemical structure database	∼ 33 million	[9]
ChemSpider	General chemical structure database	∼ 28 million	[11]
Zinc	General chemical structure database (commercially available small molecules)	∼ 20 million	[10]
Metlin	Metabolites and MS/MS data	∼ 64 000	[14]
HMDB	Human metabolome database	8608	[4]
KEGG compound	A collection of small molecules, biopolymers and other compounds relevant to biological systems	16834	[15]
DrugBank	Drugs (approved, illicit, withdrawn and experimental)	6711	[12]
PlantCyc	Plant metabolites and pathways	3334	[16]
HumanCyc	Human metabolites and pathways	1321	[13]

A typical MIMW (± 10 ppm) search in PubChem may yield several thousand chemical structures, whereas the same search in HMDB often results in less than a dozen. Both types of databases have advantages and disadvantages. Querying a focused small database such as HMDB makes identification relatively trivial if the “unknown” happens to be among the candidates. However, this approach cannot be used to identify previously unknown metabolites. On the other hand, searching a large chemical database such as PubChem greatly improves the odds of finding the unknown compound in the database. On the downside, the excessive number of potential candidates in PubChem may lead to a large number of false positives making the identification of the correct “unknown” extremely difficult. However, by applying carefully configured curation steps, the candidate list from a large database may be shortened substantially. Initial curation steps may include removing disconnected structures, eliminating charged species, clustering stereoisomers and eliminating compounds containing elements other than C, H, N, O, P and S. These curation steps alone can eliminate anywhere from 40% to 90% of candidates from the initial bin of structures matching the MIMW of the unknown.

### Special Purpose Databases

Biofluids often contain compounds that are not endogenous metabolites. Examples include drugs, drug metabolites, plant compounds and other compounds found in food. Specialized databases may be used as prescreens to exclude or identify such compounds. For example, a database containing all known drugs and their metabolites can exclude compounds coming from drugs. Also, existing databases containing plant metabolites (KEGG, PMN and others), food metabolites (HMDB) or bacterial metabolites can be screened.

## 2. Biological – Likeness Filter

Although it is trivial to eliminate candidate compounds using the 6 element filter described above (C, H, N, O, P and S) if the source of the sample is a mammalian biofluid, many synthetic compounds also contain only these 6 elements. Thus, it would be helpful to be able to determine whether a database derived candidate satisfying the 6 element filter was biochemical (and thus retained) or synthetic (and thus eliminated) based solely on its structure. Structurally similar molecules tend to have similar properties and similar molecules exert similar biological activities [17]. Using two-dimensional (2D) molecular structures of 745 *E. coli* metabolites and a variety of chemoinformatics tools, Nobeli *et al*. [18] reported the first attempt to examine the metabolome of an organism on the basis of molecular structure information. Structures were analyzed and clustered according to fingerprints (fragments describing physiochemical properties). Graph-matching algorithms for finding common substructures were employed. The authors visually examined and derived a library of 57 substructures of known metabolites, acting as scaffolds, to provide a high coverage of the metabolome. Those fragments were used to analyze the molecular composition of metabolites. Preliminary efforts of correlating the similarities between metabolites with protein structures as well as with metabolic pathways were reported. It was observed that most of the *E. coli* metabolites were hydrophilic and had molecular weights between 100 and 300 Da.

Several studies investigated statistical methods distinguishing the molecular structures of natural products, synthetic products and drugs [19–22]. Gupta and Aires-de-Sousa [23] carried out a comparative study of the chemical space occupied by metabolites versus the chemical space occupied by a diverse set of commercially available synthetic compounds. The KEGG/LIGAND database (including metabolites from different species as well as xenobiotics) was used to define the biochemical space of metabolites. The average molecular weight of this set of compounds was 311 ± 267 Da with a maximum molecular weight of 2,250 Da. The chemical space of non-metabolites was represented by a random set of commercially available compounds in the mass range 17 – 1,006 Da (average 374 ± 95 Da) from the ZINC [24] chemical database. Both chemical spaces were compared based on 2D and3D structures and descriptors of global properties. It was observed that the overlap between metabolites and non-metabolites was least in the space defined by the global descriptors. It was found that the most discriminatory features were the molecular weight, the presence of aromatic systems, and the number of OH groups. Using a random forest (RF) [25] classifier and global molecular descriptors they were able to correctly identify 95% of the 1,811 KEGG compounds used for training the model.

Peironcely *et al*. [26] extended Gupta and Aires-de-Sousa's work by using molecules in HMDB to represent endogenous human metabolites and used an updated collection of compounds from ZINC as non-biological structures. They used different molecular descriptors, such as ECFP_4 [27] and MDL public keys [28], as well as classifiers such as support vector machines [29], RF and naïve bayes [30], to evaluate the reliability of distinguishing metabolites from non-metabolites. They showed that using MDL public keys and RF resulted in the best accuracy for their classifier. The authors reported that 96% of 457 HMDB compounds, 54% of 6,532 DrugBank compounds and 22% of 6,312 compounds from ChEMBL [31] were classified as biological.

Both Gupta and Aires-de-Sousa and Peironcely *et al*. employed fingerprints [32] for classification. Finding common substructures has the potential to describe structural similarity more accurately than fingerprint-based methods but it is much slower [18]. With the current advances in technology such as threading and the use of multiple cores this is no longer a significant limitation.

Recently, Hamdalla *et al*. [33] developed a cheminformatics tool that utilizes the molecular structures of known human metabolites to identify potential unknowns from a list of candidate structures. It uses a graph matching tool (SMSD [34]) and a curated set of 1,400 endogenous human metabolites from KEGG (scaffolds) to guide its classification process. This process is based on a scoring scheme that combines all matches of scaffolds to substructures of a candidate compound as well as matches of the candidate compound to substructures of the scaffolds. Preliminary results using leave-one-out cross validation experiments showed that 96% of 1,400 KEGG endogenous human metabolites were scored as biological. However, when a set of synthetic chemical compounds obtained from Chembridge [35] and Chemsynthesis [36] databases were examined, 46% of 5,320 structures were predicted to be biological. Hence, this approach allows the user to search large chemical databases, but removes a significant number of synthetic chemical compounds from the final candidate list.

## 3. Quantitative Structure Property Relationship (QSPR) Models

Quantitative structure property relationship (QSPR) based models can be used to predict physiochemical properties of compounds in databases. QSPR models relate measurements of a set of predictor variables to a response variable via a regression procedure [37]. In QSPR, the predictor variables comprise properties of chemicals in the form of molecular descriptors. Often, a molecular descriptor is a theoretical value derived from a symbolic representation of the chemical structure. The response variable can be an experimentally derived property such as retention index. Regression procedures used in QSPR models range from simple multiple linear correlations to non-linear models such as artificial neural networks (ANN) and random forests (RF). The physiochemical properties chosen for HPLC/MS based QSPR modeling might include HPLC retention index (RI), ECOM_50_ and drift time. Candidate compounds whose predicted values deviate substantially from the experimental value are excluded from the final candidate list.

### 3.1 Retention Index

Chromatographic retention times are frequently used as an aid in characterizing compounds. In HPLC, analytes dissolved in a mobile phase are moved across a stationary phase. The relative affinity of the analyte (via non-covalent interactions) between the mobile phase and the stationary phase determines the amount of analyte retention. Since non-covalent interactions between the analyte and the mobile and stationary phases are a function of structure, even subtle changes in structure can result in well-separated analytes. However, factors such as small variations in the composition of the mobile phase, the pH of the eluent and even temperature can alter the retention time, thus making comparisons over time and between instruments difficult. To alleviate this problem, retention indices are often used. The retention index is a measure of the retention time relative to a homologous series of compounds such as *n-*nitroalkanes [7, 8, 38, 39]. In this approach, retention time is converted to a retention index by comparing the number of carbons in the standard that elutes just before and just after the analyte. HPLC retention indices are shown to be quite robust with a high degree of reproducibility within a single instrument over a long period of time as well as between different instruments [7, 38–40].

QSPR models have been developed for predicting HPLC retention times and retention indices based on molecular structure. Moon *et al*. modeled HPLC retention times for a set of poly aromatic hydrocarbon compounds using one and two descriptor multiple linear regression (MLR) models [41]. Several molecular descriptors were tested including molecular weight, molecular connectivity, length to breadth ratio, highest occupied and lowest unoccupied molecular orbital energies, volume, Connolly surface area and dipole moment using MOPAC software package. The authors found several two descriptor models that show a good correlation with the retention time. The best predictive model included molecular connectivity and dipole moment as descriptors.

Ghosh *et al*. developed a partial least squares based quantitative model for predicting HPLC retention times of various aromatic and poly aromatic hydrocarbons [42]. Forty-four aromatic compounds containing one to three ring structures were used in the QSPR model. Molecular descriptors were calculated using the program CAChe. Geometry optimizations were carried out using the semi empirical method PM3 as implemented in program MOPAC. It was found that both electronic and geometric descriptors play a vital role in determining the retention time of a molecule. The most significant molecular descriptors included ionization potential, electron affinity, molecular weight, valence connectivity index of order 1, valence connectivity index of order 2 and number of rings. The authors found a good agreement between the predicted and experimental retention times with correlation coefficients of 0.905 in the training set and 0.831 in the testing set.

Albaugh *et al*. reported the first retention index model suitable for predicting HPLC retention indices of unknown compounds in complex mixtures [39]. The authors developed two predictive models based on MLR and ANN using a diverse set of drug-like compounds. The dataset contained 498 compounds with structures ranging from simple aromatic hydrocarbons to compounds containing a large number of heteroatoms and fused ring systems. The QSPR models were built using a novel set of descriptors called interaction groups (IGroup). These descriptors combine atomic E-state descriptors, which encode the electron accessibilities of individual atoms in molecules. The IGroup descriptors can be regarded as a variation of functional group type E-state indices that combine E-states of atoms in similar functional groups. Several other global descriptors related to molecular connectivity, volume and polar surface area were also used in the models. The MLR model showed a minimally acceptable correlation coefficient of 0.65 in the training set, 0.45 in the cross validation set and 0.49 in the external validation set. The mean absolute error (MAE) for the MLR model was 83.6 RI units in the training set, 83.5 RI units in the cross validation set and 79.5 RI units in the external validation set. The ANN model showed much better correlation coefficients; 0.93 in the training set, 0.76 in the cross validation set and 0.83 in the external validation set. The MAEs were 30.3, 53.7 and 40.8 RI units for the training, cross validation and external validation sets respectively. One limitation of retention index predictive models is the specificity of the model to the type of column and mobile phase used in the experiment. Therefore, a change in mobile phase or column type will require development of a new predictive model. The predictive model developed in the Albaugh *et al*. study is not suitable for HPLC/MS as the mobile phase used is not compatible with electrospray ionization mass spectrometry.

In a recent study, Hall *et al*. reported an ANN based retention index model suitable for use in HPLC/MS applications [7]. The model was developed using 33 Molconn structure descriptors. Four hundred endogenous and drug-like compounds were used in the training of the neural network model. The predictive model had correlation coefficients of 0.95 in the training set, 0.83 in the cross validation set and 0.87 in the external validation set. The MAE for the RI model was 19 RI units in the training set, 36 RI units in the cross validation set and 30 RI units in the external validation set. Ninety percent of cross validation predictions and ninety three percent of external validation predictions were within 75 RI units of the measured retention index. The authors were able to use the RI model developed in this study as an aid in identifying 1,3-dicyclohexylurea in human plasma. This compound was not previously known to exist in human biofluids and was not found in any of the biological databases.

### 3.2 ECOM_50_

In CID mass spectrometry, an accelerated molecular ion is allowed to collide with inert gas molecules such as argon or nitrogen. Upon collision, some of the kinetic energy of the accelerated ion is converted into internal energy. The absorbed energy is redistributed throughout the molecule via molecular vibrations. When the energy absorbed by the molecular ion exceeds a certain threshold, molecular vibrations cause the ion to dissociate. This suggests that the external energy (collision energy) required to start fragmentation is a unique property of the structure and can be used as a parameter for identifying unknown compounds. Previous survival yield analysis studies have shown that there exists a sigmoidal relationship between the amount of intact precursor ion and collision energy, and that the collision energy at which 50% of the precursor ion remains intact (CE_50_) is a unique and highly reproducible quantity [43]. Furthermore, it has been shown that CE_50_ values are independent of cone potential and orthogonal to exact mass. A preliminary study by Kertesz *et al*. showed that it is possible to discriminate among similar structural isomers using CE_50_ [43]. The authors measured CE_50_ values for seven isomers (including three positional isomers) of the molecular formula C_9_H_11_NO_2_. The CE_50_ values ranged from 8.24 to 16.52 with significantly different values for three positional isomers. In the same study, a QSPR model was developed for predicting CE_50_ values. The MLR based model comprised eight E-state descriptors and showed a correlation coefficient of 0.81.

In addition to the structure of the molecular ion, the type of collision gas used also affects the CE_50_. A collision gas independent form of CE_50_ can be obtained by using the following formula:

$$ECOM_{50}=(CE_{50}\times M_{rg})/(M_{rg}+M_{x})$$

where, ECOM_50_ is the center of mass energy at 50% survival yield and M_rg_ and M_x_ are the MIMWs of reagent gas and analyte molecular ion respectively.

A QSPR model for predicting ECOM_50_ would be most useful if the measured values are comparable between different instruments. In a recent study, Hill *et al*. investigated the influence of physical and electrical characteristics of different mass spectrometry instruments on ECOM_50_ [44]. The ECOM_50_ values measured on four different instruments were highly correlated, with correlation coefficients that ranged from 0.953 to 0.992. However, the authors suggested caution when comparing ECOM_50_ values (and CID spectra) measured on different instruments without correcting for ion transfer efficiencies. Hall *et al*. developed several ECOM_50_ models using MLR and partial least squares (PLS) methods [7]. The dataset used to construct ECOM_50_ models comprised 52 compounds covering the mass range from 88.2 to 607.7 Da. Separate models were developed for neutral and singly protonated forms of the training data. Two types of models were developed using Molconn topological descriptors and AMPAC-CODESSA quantum mechanical descriptors. Correlation coefficients for the Molconn based models ranged from 0.848 (neutral structures with MLR) to 0.931 (protonated structures with PLS) depending on the type of algorithm chosen and whether protonated or neutral forms were used. The MLR based AMPAC-CODESSA models also showed a good correlation with a correlation coefficient of 0.920 for the neutral structure model and 0.943 for the singly protonated structure model. In general, the use of singly protonated structures resulted in improved predictions although the improvement was not significant for CODESSA models. The ECOM_50_ models developed in this study should be considered as preliminary models because the data set used for training lacked certain types of chemical functional groups. Despite these limitations, the authors were able to use the protonated structure based Molconn PLS model to filter out 10% of compounds from a PubChem bin leading to the identification of a previously unknown metabolite 1,3-dicyclohexylurea.

### 3.3 Drift Time

Ion mobility spectrometry [45, 46] is a molecular shape based separation method where compounds are separated by the time (drift time) a compound takes to traverse a gas-filled cell under the influence of an external electric field. When coupled with mass spectrometry, it allows for the separation of ions with identical m/z values. Ion mobility-mass spectrometry (IMMS) is used as a technique for discriminating closely related structures such as enantiomers [47], diastereomers [48], protein conformers [49, 50] and isomeric drug metabolites [51]. In conventional drift tube based IMMS, the drift time is proportional to the average collisional cross sectional area of the gas phase ion. Structure identification often involves comparing experimentally derived cross sectional areas with theoretically calculated cross sectional areas. However, conventional IMMS is limited by its low ion transfer efficiency. This shortcoming has been addressed in modern travelling wave (T-Wave) based Synapt IMMS systems [52]. In T-Wave instruments, the drift time shows a power-law relationship with cross sectional area due to the complex electric field used [53]. An experimentally derived cross sectional area comparable to that of a conventional drift tube can be obtained by calibrating a T-wave instrument with poly alanine standards [51, 54, 55].

The open source program Mobcal [56, 57] is often used for theoretical cross sectional area calculations. Mobcal calculates theoretical cross sectional area by three methods: projection approximation (PA), exact hard sphere scattering approximation (EHSS) and trajectory method (TM). The trajectory method (with optimized parameters for a given drift gas) has shown to be the most accurate of the three for small to medium and fairly rigid molecules [48, 51]. However, Mobcal cross sectional areas of large and flexible molecules deviate substantially from experimental values [58]. The errors often point to inaccuracies associated with the optimized molecular structures. In many cases, optimizing the starting geometry with high level theory (using high level quantum chemistry methods with a larger basis set) or using an ensemble of starting geometries instead of a single geometry did not improve calculated cross sectional areas. In a recent study [8], we proposed an alternative method using QSPR models. In this approach, molecular descriptors were used to compensate for inaccuracies associated with the starting geometries. In addition, descriptors provide a way to include characteristics of flexible molecules. The RF based models developed in this study outperformed the widely used Mobcal trajectory (with optimized parameters for N_2_) method.

## 4. Computational Prediction of CID Spectra

Ranking candidate compounds based on CID spectra matching is the final step of the identification process. In this step, a computationally predicted spectrum is matched against an experimentally observed spectrum using either the number of peaks matching or a score, which may include intensity information and bond energies in addition to matched peaks. CID prediction algorithms can be broadly categorized into 2 groups: rule based and combinatorial fragmentation based. Rule based algorithms use a set of known decomposition reactions from the literature. The decomposition reactions may include generic McLafferty type rules [59], a library of structure specific reactions or a combination of both. Mass Frontier [60] and ACD/MS Fragmenter [61] are examples for programs using rule-based methods. Some of the limitations of rule-based methods include over prediction due to broadly generic rules, lack of specific rules for certain types of compounds and slowness in the library search mode. Combinatorial fragmenters such as FiD [62] and MetFrag [63] use a simple bond disconnection approach. One downside to this approach is not being able to account for rearrangement products. Previous studies using both approaches have shown both to be effective in identifying unknowns [5, 7, 63]. A previous study by Hill *et al*. identified 65 out of 102 compounds using Mass Frontier (version 4.0) peaks matching [5]. The surrogate unknown was found within the top 20 candidates for 87 bins. Wolf *et al*. were able to achieve a slightly better result than Hill *et al*. using the same dataset, but with MetFrag [63]. Our own study [8] suggests advantages and disadvantages of both approaches; where one might work better than the other on a case-by-case basis.

## Summary and Outlook

Database searching and filtering offers an alternative to identifying unknowns using purified standards. In addition, it complements authentic standards based identification techniques by providing a short list of potential standards to experimentally compare to the unknown. Current RI and ECOM_50_ models allow for the removal of 28% of compounds from PubChem bins [8]. In a recent study, we have shown that this could be improved to as much as 87% with more chemical structures in the QSPR models [8]. In addition to RI and ECOM_50_, drift time can also be used as a potential filter. The drift time model was shown to be quite effective for large molecular weight bins containing compounds with more flexible structures. Furthermore, the entire workflow can be executed in an automated fashion using the program MolFind [8]. We expect that filtering and identification of metabolites will be much more reliable and efficient with improved computational models.

## References

[1] Wishart DS (2011) Advances in metabolite identification. Bioanalysis 3: 1769–1782 21827274 10.4155/bio.11.155

[2] Wishart DS (2007) Current progress in computational metabolomics. Brief Bioinform 8: 279–293 17626065 10.1093/bib/bbm030

[3] Oliver SG, Winson MK, Kell DB, Baganz F (1998) Systematic functional analysis of the yeast genome Trends Biotechnol 16: 373–378 10.1016/s0167-7799(98)01214-1 9744112

[4] Wishart DS, Tzur D, Knox C, Eisner R, Guo AC, et al (2007) HMDB: the Human Metabolome Database. Nucleic Acids Res 35: D521–526 17202168 10.1093/nar/gkl923 PMC1899095

[5] Hill DW, Kertesz TM, Fontaine D, Friedman R, Grant DF (2008) Mass spectral metabonomics beyond elemental formula: chemical database querying by matching experimental with computational fragmentation spectra. Anal Chem 80: 5574–5582 18547062 10.1021/ac800548g

[6] Kertesz TM, Hill DW, Albaugh DR, Hall LH, Hall LM, et al (2009) Database searching for structural identification of metabolites in complex biofluids for mass spectrometry-based metabonomics. Bioanalysis 1: 1627–1643 21083108 10.4155/bio.09.145

[7] Hall LM, Hall LH, Kertesz TM, Hill DW, Sharp TR, et al (2012) Development of Ecom(50) and Retention Index Models for Nontargeted Metabolomics: Identification of 1,3-Dicyclohexylurea in Human Serum by HPLC/Mass Spectrometry. J Chem Inf Model 52: 1222–1237 22489687 10.1021/ci300092s PMC3376006

[8] Menikarachchi LC, Cawley S, Hill DW, Hall LM, Hall L, et al (2012) MolFind: A Software Package Enabling HPLC/MS-Based Identification of Unknown Chemical Structures. Anal Chem 84: 9388–93394 23039714 10.1021/ac302048x PMC3523192

[9] Bolton EE, Wang Y, Thiessen PA, Bryant SH (2008) PubChem: Integrated Platform of Small Molecules and Biological Activities. Annual Reports in Computational Chemistry. Washington, DC: American Chemical Society, Vol. 4 pp. 217–241

[10] Irwin JJ, Sterling T, Mysinger MM, Bolstad ES, Coleman RG (2012) ZINC: A Free Tool to Discover Chemistry for Biology. J Chem Inf Model 52: 1757–1768 22587354 10.1021/ci3001277 PMC3402020

[11] Chemspider (2012) http://www.chemspider.com.

[12] Knox C, Law V, Jewison T, Liu P, Ly S, et al (2011) DrugBank 3.0: a comprehensive resource for “omics” research on drugs. Nucleic Acids Res 39: D1035–41 21059682 10.1093/nar/gkq1126 PMC3013709

[13] Romero P, Wagg J, Green ML, Kaiser D, Krummenacker M, et al (2005) Computational prediction of human metabolic pathways from the complete human genome. Genome Biol 6: R2 15642094 10.1186/gb-2004-6-1-r2 PMC549063

[14] Smith C a, O'Maille G, Want EJ, Qin C, Trauger S a, et al (2005) METLIN: a metabolite mass spectral database. Ther Drug Monit 27: 747–751 16404815 10.1097/01.ftd.0000179845.53213.39

[15] Kanehisa M, Goto S (2000) KEGG: kyoto encyclopedia of genes and genomes. Nucleic Acids Res 28: 27–30 10592173 10.1093/nar/28.1.27 PMC102409

[16] Chae L, Lee I, Shin J, Rhee SY (2012) Towards understanding how molecular networks evolve in plants. Curr Opin Plant Biol 15: 177–184 22280840 10.1016/j.pbi.2012.01.006

[17] Maggiora GM, Shanmugasundaram V (2011) Molecular similarity measures. Methods in molecular biology (Clifton, NJ) 672: 39–100 10.1007/978-1-60761-839-3_2 20838964

[18] Nobeli I, Ponstingl H, Krissinel EB, Thornton JM@ (2003) A Structure-based Anatomy of the E.coli Metabolome J Mol Biol 334: 697–719. 10.1016/j.jmb.2003.10.008 14636597

[19] Henkel T, Brunne RM, Müller H, Reichel F (1999) Statistical Investigation into the Structural Complementarity of Natural Products and Synthetic Compounds. Angew Chem Int Ed 38: 643–647 10.1002/(SICI)1521-3773(19990301)38:5<643::AID-ANIE643>3.0.CO;2-G 29711552

[20] Lee M-L, Schneider G (2001) Scaffold Architecture and Pharmacophoric Properties of Natural Products and Trade Drugs: Application in the Design of Natural Product-Based Combinatorial Libraries. J Comb Chem 3: 284–289 11350252 10.1021/cc000097l

[21] Koch M a, Schuffenhauer A, Scheck M, Wetzel S, Casaulta M, et al (2005) Charting biologically relevant chemical space: a structural classification of natural products (SCONP). Proc Natl Acad Sci U S A 102: 17272–17277 16301544 10.1073/pnas.0503647102 PMC1297657

[22] Ortholand J-Y, Ganesan A (2004) Natural products and combinatorial chemistry: back to the future. Curr Opin Chem Biol 8: 271–280 15183325 10.1016/j.cbpa.2004.04.011

[23] Gupta S, Aires-de-Sousa J (2007) Comparing the chemical spaces of metabolites and available chemicals: models of metabolite-likeness. Mol Diversity 11: 23–36 10.1007/s11030-006-9054-0 17447158

[24] Irwin JJ, Shoichet BK (2005) ZINC-a free database of commercially available compounds for virtual screening. J Chem Inf Model 45: 177–182 15667143 10.1021/ci049714 PMC1360656

[25] Breiman L (2001) Random forests Machine Learning 45: 5–32

[26] Peironcely JE, Reijmers T, Coulier L, Bender A, Hankemeier T (2011) Understanding and classifying metabolite space and metabolite-likeness. PLoS One 6: e28966 22194963 10.1371/journal.pone.0028966 PMC3237584

[27] Rogers D, Hahn M (2010) Extended-connectivity fingerprints. J Chem Inf Model 50: 742–754 20426451 10.1021/ci100050t

[28] Durant J, Leland B, Henry DR, Nourse JG (2002) Reoptimization of MDL Keys for Use in Drug Discovery. J Chem Inf Model 42: 1273–1280 10.1021/ci010132r 12444722

[29] Noble WS (2006) What is a support vector machine?. Nat Biotechnol 24: 1565–1567 17160063 10.1038/nbt1206-1565

[30] Klon AE, Glick M, Davies JW (2004) Combination of a naive Bayes classifier with consensus scoring improves enrichment of high-throughput docking results. J Med Chem 47: 4356–4359 15317449 10.1021/jm049970d

[31] Overington J (2009) ChEMBL. An interview with John Overington, team leader, chemogenomics at the European Bioinformatics Institute Outstation of the European Molecular Biology Laboratory (EMBL-EBI). Interview by Wendy A. Warr. J Comput-Aided Mol Des 23: 195–198 10.1007/s10822-009-9260-9 19194660

[32] James CA, Weininger D, Delany J (2000) Fingerprints - Screening and Similarity Daylight Theory Manual. Irvine, CA and Santa Fe, NM: Daylight Chemical Information Systems, Inc pp. 30–40

[33] Hamdalla M, Grant D, Mandoiu I, Hill D, Rajasekaran S et al (2012) The use of graph matching algorithms to identify biochemical substructures in synthetic chemical compounds: Application to metabolomics. 2012 IEEE 2nd International Conference on Computational Advances in Bio and medical Sciences (ICCABS). IEEE pp. 1–6 10.1109/ICCABS.2012.6182637 PMC4593623 26448899

[34] Rahman SA, Bashton M, Holliday GL, Schrader R, Thornton JM (2009) Small Molecule Subgraph Detector (SMSD) toolkit. Journal of Cheminformatics 1: 12 20298518 10.1186/1758-2946-1-12 PMC2820491

[35] Chembridge (2011) http://www.chembridge.com/.

[36] Chemsynthesis (2011) http://www.chemsynthesis.com/.

[37] Leach A, Gillet V(2007) An introduction to chemoinformatics Springer pp. 75–97

[38] Hill DW, Kind AJ (1994) Reversed-Phase Solvent-Gradient HPLC Retention Indexes of Drugs. Journal of Analytical Toxicology 18: 233–242 7990438 10.1093/jat/18.5.233

[39] Albaugh DR, Hall LM, Hill DW, Kertesz TM, Parham M (2009) Prediction of HPLC retention index using artificial neural networks and IGroup E-state indices. J Chem Inf Model 49: 788–799 19309176 10.1021/ci9000162

[40] Bogusz M, Neidl-Fischer G, Aderjan R (1988) Use of Corrected Retention Indices Based on 1-Nitroalkane and Alkyl Arylketone Scales for HPLC Identification of Basic Drugs. J Anal Toxicol 12: 325–329 2907596 10.1093/jat/12.6.325

[41] Moon T, Whan Chi M, Ja Park S, No Yoon C (2003) Prediction of HPLC Retention Time Using Multiple Linear Regression: Using One and Two Descriptors. J Liq Chromatogr Relat Technol 26: 2987–3002

[42] Ghosh P, Chawla B, Joshi P V., Jaffe SB (2006) Prediction of Chromatographic Retention Times for Aromatic Hydrocarbons Energy Fuels 20: 609–619

[43] Kertesz TM, Hall LH, Hill DW, Grant DF (2009) CE50: quantifying collision induced dissociation energy for small molecule characterization and identification. J Am Soc Mass Spectrom 20: 1759–1767 19616966 10.1016/j.jasms.2009.06.002

[44] Hill DW, Baveghems CL, Albaugh DR, Kormos TM, Lai S, et al (2012) Correlation of Ecom50 values between mass spectrometers: effect of collision cell radiofrequency voltage on calculated survival yield Rapid Commun Mass Spectrom 26: 2303–2310 10.1002/rcm.6353 PMC3439163 22956322

[45] Kanu AB, Dwivedi P, Tam M, Matz L, Hill HH (2008) Ion mobility-mass spectrometry. J Mass Spectrom 43: 1–22 18200615 10.1002/jms.1383

[46] Creaser CS, Griffiths JR, Bramwell CJ, Noreen S, Hill CA, et al (2004) Ion mobility spectrometry: a review. Part 1. Structural analysis by mobility measurement The Analyst 129: 984–994

[47] Dwivedi P, Wu C, Matz LM, Clowers BH, Siems WF et al (2006) Gas-phase chiral separations by ion mobility spectrometry. Anal Chem 78: 8200–8206 17165808 10.1021/ac0608772 PMC3633475

[48] Campuzano I, Bush MF, Robinson C V, Beaumont C, Richardson K, et al (2012) Structural Characterization of Drug-like Compounds by Ion Mobility Mass Spectrometry: Comparison of Theoretical and Experimentally Derived Nitrogen Collision Cross Sections. Anal Chem 84: 1026–1033 22141445 10.1021/ac202625t

[49] Smith DP, Giles K, Bateman RH, Radford SE, Ashcroft AE (2007) Monitoring copopulated conformational states during protein folding events using electrospray ionization-ion mobility spectrometry-mass spectrometry. J Am Soc Mass Spectrom 18: 2180–2190 17964800 10.1016/j.jasms.2007.09.017 PMC2706321

[50] Valentine SJ, Clemmer DE (1997) H/D Exchange Levels of Shape-Resolved Cytochrome c Conformers in the Gas Phase. J Am Chem Soc 119: 3558–3566

[51] Dear GJ, Munoz-Muriedas J, Beaumont C, Roberts A, Kirk J, et al (2010) Sites of metabolic substitution: investigating metabolite structures utilising ion mobility and molecular modelling Rapid Commun Mass Spectrom 24: 3157–3162 10.1002/rcm.4742 20941763

[52] Giles K, Williams JP, Campuzano I(2011) Enhancements in travelling wave ion mobility resolution. Rapid Communications in Mass Spectrometry : RCM 25: 1559–1566 21594930 10.1002/rcm.5013

[53] Smith DP, Knapman TW, Campuzano I, Malham RW, Berryman JT, et al (2009) Deciphering drift time measurements from travelling wave ion mobility spectrometry-mass spectrometry studies. Eur J Mass Spectrom 15: 113–130 10.1255/ejms.947 19423898

[54] Williams JP, Bugarcic T, Habtemariam A, Giles K, Campuzano I, et al (2009) Isomer separation and gas-phase configurations of organoruthenium anticancer complexes: ion mobility mass spectrometry and modeling. J Am Soc Mass Spectrom 20: 1119–1122 19297193 10.1016/j.jasms.2009.02.016

[55] Kim HI, Kim H, Pang ES, Ryu EK, Beegle LW, et al (2009) Structural characterization of unsaturated phosphatidylcholines using traveling wave ion mobility spectrometry. Anal Chem 81: 8289–8297 19764704 10.1021/ac900672a PMC2761977

[56] Shvartsburg A (1996) An exact hard-spheres scattering model for the mobilities of polyatomic ions. Chem Phys Lett 261: 86–91

[57] Mesleh MF, Hunter JM, Shvartsburg AA, Schatz GC, Jarrold MF (1996) Structural Information from Ion Mobility Measurements: Effects of the Long-Range Potential. J Phys Chem 100: 16082–16086

[58] Zakharova NL, Crawford CL, Hauck BC, Quinton JK, Seims WF, et al (2012) An assessment of computational methods for obtaining structural information of moderately flexible biomolecules from ion mobility spectrometry. J Am Soc Mass Spectrom 23: 792–805 22359091 10.1007/s13361-012-0339-5

[59] McLafferty FW (1980) Unimolecular decompositions of even-electron ions. Org Mass Spectrom 15: 114–121

[60] Mass Frontier 7.0 (2012) http://www.thermoscientific.com.

[61] ACD/MS Fragmenter (2012) http://www.acdlabs.com.

[62] Heinonen M, Rantanen A, Mielikäinen T, Kokkonen J, Kiuru J, et al (2008) FiD: a software for ab initio structural identification of product ions from tandem mass spectrometric data Rapid Commun Mass Spectrom 22: 3043–3052 10.1002/rcm.3701 18763276

[63] Wolf S, Schmidt S, Müller-Hannemann M, Neumann S (2010) In silico fragmentation for computer assisted identification of metabolite mass spectra. BMC Bioinf 11: 148 10.1186/1471-2105-11-148 PMC2853470 20307295

